# Safety Assessment of Cassava Pulp-Derived Dietary Fiber: Acute and Sub-Acute Toxicity Evaluation

**DOI:** 10.3390/toxics13060473

**Published:** 2025-06-03

**Authors:** Naiyana Nontamart, Kakanang Posridee, Parin Suwannaprapha, Rungrudee Srisawat, Ratchadaporn Oonsivilai

**Affiliations:** 1 School of Preclinical Science, Institute of Science, Suranaree University of Technology, Nakhon Ratchasima 30000, Thailand; anitech10@gmail.com; 2 Health and Wellness Research Unit, School of Food Technology, Institute of Agricultural Technology, Suranaree University of Technology, Nakhon Ratchasima 30000, Thailand; posridee.ka@gmail.com; 3 Department of Pre-Clinic and Applied Animal Science, Faculty of Veterinary Science, Mahidol University, Nakhon Pathom 73170, Thailand; parin.suw@mahidol.edu

**Keywords:** acute toxicity, sub-acute toxicity, dietary fiber, cassava pulp, Wistar rat

## Abstract

This study rigorously evaluated the safety profile of dietary fiber extracted from cassava pulp, a promising functional food ingredient, through acute and 28-day sub-acute oral toxicity assessments in Wistar rats. This research hypothesized that cassava pulp fiber would exhibit minimal toxicity across a range of doses. In the acute study, rats received single oral doses of 175, 550, or 2000 mg/kg, while the sub-acute toxicity study involved daily doses of 250, 500, or 1000 mg/kg, with satellite groups included for reversibility assessment. Comprehensive monitoring encompassed clinical signs, mortality, body weight, food intake, hematological and biochemical parameters, relative organ weights, and detailed histopathological examination. Remarkably, no treatment-related mortality or overt clinical signs of toxicity were observed in either study. The LD_50_ was higher than 2000 mg/kg for the acute study and the no-observed-adverse-effect level (NOAEL) was determined to be 2000 mg/kg for the acute study and 1000 mg/kg for the sub-acute toxicity study, indicating a high margin of safety. While statistically significant alterations were noted in some hematological, biochemical, and relative organ weight parameters, these changes were not considered toxicologically relevant. Notably, histopathological changes in the lungs were observed across all groups, including controls, warranting further investigation. These findings suggest that cassava pulp fiber is well tolerated at high oral doses, supporting its potential for safe application in food and nutraceutical formulations. However, the observed lung alterations necessitate further research to elucidate their etiology and clinical significance.

## 1. Introduction

Cassava waste is classified as a raw material, a residue from industrial factories that produce cassava starch. The primary effluent from cassava processing, specifically the pulp fraction, exhibits a high moisture content, typically around 80%. This wet byproduct constitutes a significant feed source for livestock, including cattle and buffaloes, as well as for aquaculture. Conversely, the dried cassava pulp is often relegated to a lower-value application as an additive in the production of cassava chips or pellets, which consequently reduces the overall quality of these products. Despite this secondary utilization, cassava pulp retains substantial nutritional value, particularly in terms of readily digestible non-fiber carbohydrates (NFE), which comprise approximately 65–70% of its dry matter. The nutritional value of dried cassava pulp contains protein, calcium, fiber, phosphorus, and fat at 1.83%, 0.60%, 10%, 0.36%, and 0.48% (*w*/*w*), respectively. There is still quite a lot of flour remaining [1]. In general, these wastes can be made into fertilizer and animal food. At present, it is still commonly used as an alternative energy source, which is receiving a lot of attention due to the situation of the world’s population looking for other energy sources to compensate for the oil energy source, which is decreasing day by day. But one interesting option is to process cassava waste into other raw materials, in addition to the above-mentioned options. Knowledge of science and food technology can be used to process cassava waste into substances with various properties. It can be used in both the food and pharmaceutical industries.

Dietary fiber is the part of plants, vegetables, and fruits that people can eat. It is not digested by human digestive juices but may be digested by some microorganisms in the human digestive tract. Dietary fiber is a large molecule of carbohydrate. It is beneficial to the body in terms of facilitating bowel movements. It helps capture fat from food to reduce the absorption of sugar-type substances. Therefore, it has a beneficial effect on patients with diabetes. It helps prevent the absorption of carcinogens because it can be excreted quickly and reduce contact with the intestinal wall. Dietary fiber also helps in weight loss. Increased dietary fiber intake promotes water absorption within the digestive tract, leading to enhanced satiety and a subsequent reduction in overall food consumption. Furthermore, the water-holding capacity of cellulose-rich dietary fiber significantly influences fecal characteristics, resulting in softer stools and facilitating regular bowel movements, thereby mitigating constipation. These physiological effects are implicated in the prevention of anorectal conditions such as hemorrhoids, as well as potentially reducing the risk of intestinal diverticular disease and colorectal cancer [2].

The influence of neutral detergent fiber (NDF) on mercury bioavailability was investigated using an integrated in vitro digestion and Caco-2 human intestinal cell model system. In vitro digestion assays revealed a dose-dependent reduction in mercury bio-accessibility (2–57%) in the presence of NDF (0–1000 mg NDF per 1 g fish tissue) compared to the control. Furthermore, when 500 mg of NDF was incorporated into the digestion model with 0.4 g of fish tissue, mercury quantification indicated a decrease in mercury content within the fish tissue from 39% to 21% relative to the NDF-free control. To assess intestinal cell accumulation and provide a robust estimation of bioavailability, Caco-2 cells were employed. The results demonstrated a reduction in mercury transfer to the intracellular compartment from 9.07–5.97% in the control group to 6.54% in the presence of 500 mg NDF in the media. Collectively, these findings suggest that NDF derived from cassava pulp possesses the capacity to diminish mercury bioavailability by impeding its transfer to the aqueous phase during digestion [3].

Short-term toxicity assessment and evaluation of potential hypocholesterolemia effects of cassava pulp dietary fiber (CDF) revealed that CDF exhibits good tolerability, with NOAEL established at 10.01 g/kg body weight/day for male Sprague–Dawley rats and 11.21 g/kg body weight/day for female Sprague–Dawley rats. Furthermore, dietary supplementation with CDF in Wistar rats fed a high-fat diet resulted in a statistically significant reduction in cholesterol levels (*p* < 0.05) when compared to the effects of simvastatin [4].

Despite cassava pulp’s established status as a substantial source of dietary fiber, a critical gap exists in the comprehensive understanding of its acute and sub-acute toxicological profiles. This study addresses this deficiency by investigating the potentially toxic effects of dietary fiber derived from cassava pulp in a Wistar rat model.

## 2. Materials and Methods

### 2.1. Materials and Sample Preparation

Cassava pulp was obtained from Sanguan Wongse Industries Co., Ltd., in Nakhon Ratchasima Province, Thailand. Key enzymes utilized included heat-stable α-amylase (Termamyl 120 L, EC 3.2.1.1, Merck, Darmstadt, Germany), amyloglucosidase (AMG 300 L from Aspergillus niger, EC 3.2.1.3, Bray, Co., Wicklow, Ireland), and neutrase^®^ (EC 3.4.24.28 from Bacillus amyloliquefaciens, Novozymes Co., Bagsvaerd, Denmark). All other chemicals were of reagent grade. A standard rat diet (082G/15, C.P. mouse feed, Perfect Companion Group, Bangkok, Thailand) served as the control.

Prior to use, the cassava pulp was dried at 60 °C for 24 h using a tray dryer (Kluaynumtaitowop, Bangkok, Thailand). The dried material was then finely ground (GmbH & Co., KG D-42781, Haan, Germany) and stored in a vacuum-packed container at room temperature.

### 2.2. Dietary Fiber Preparation

Dietary fiber from cassava pulp (CDF) was prepared following established methodologies outlined in previous research [3,4]. A 4% (*w*/*v*) solution of cassava pulp in 50 mM phosphate buffer (pH 6) was prepared. This solution was first treated with 0.1% (*w*/*v*) heat-stable α-amylase (Termamyl, Merck, Darmstadt, Germany) at 95 °C and pH 6 for 30 min. The pH was then adjusted to 7.5 using 0.17 M sodium hydroxide (Merck Ltd., Darmstadt, Germany) before the addition of neutrase (1% *v*/*v*). Neutrase treatment proceeded for 30 min at 60 °C, after which the pH was lowered to 4.5 with 0.205 M phosphoric acid (Carlo). Subsequently, 0.1% (*v*/*v*) amyloglucosidase was introduced, and the mixture was treated for 30 min at 95 °C.

The resulting hydrolysate was centrifuged (Hettich, Universal 32R, DJB Labcare Ltd., Newport Pagnell, UK) at 10,000× *g* for 10 min to separate the fiber-enriched sediment from the supernatant. The sediment was then washed with distilled water, subjected to a second centrifugation at 10,000× *g* for 10 min, and finally dried at 60 °C in a hot air oven. The prepared dietary fiber powder was stored at 4 °C in a sealed container until further use.

### 2.3. Animals

Healthy male and female Wistar rats were purchased from the experimental animal department, Suranaree University of Technology, Nakhon Ratchasima Province, Thailand. The body weights ranged from 165 to 200 g for males and 120 to 160 g for females. The test was carried out following guideline no. 423: acute oral toxicity—acute toxic class method of the OECD guidelines for the testing of chemicals (2001) [5] with a modification in terms of increasing doses. Sub-chronic toxicity was studied according to the Organization of Economic Co-operation and Development (OECD) guideline 407 [OECD, 2008] for testing of chemicals: repeated dose 28-day oral toxicity study in rodents (OECD, 2008) [6].

### 2.4. Preparation of Animals (Acute Toxicity)

Following a 7-day acclimatization period and a 16–18 h fast, experimental rats were administered test substances according to pre-defined groups. Animals were then monitored for clinical signs of toxicity at 0.5, 1, 2, and 4 h post-administration, and daily for 14 days, with mortality recorded to determine LD_50_. Animals exhibiting severe pain or distress were euthanized, and post-mortem samples were collected. Throughout the study, food intake and body weight were recorded daily and weekly, respectively. On day 14, animals were euthanized via carbon dioxide inhalation, and blood and organ samples were collected for hematological, biochemical, and histopathological analyses.

### 2.5. Acute Toxicity Study

A total of 40 six-week-old Wistar rats (20 males, initial body weight 165–200 g; 20 females, initial body weight 120–160 g) were utilized in this study. Animals were randomly assigned to treatment groups (*n* = 5 per group) and received a single dose of dietary fiber derived from cassava pulp via oral gavage. The experimental design employed a factorial approach, stratifying subjects by sex and then randomly assigning them to one of four treatment groups: a control group receiving propylene glycol (10 mL/kg) or groups receiving CDF at dosages of 175, 550, or 2000 mg/10 mL/kg, respectively, thus resulting in a total of eight distinct experimental groups.

### 2.6. Sub-Acute Toxicity Study

To evaluate the impact of cassava pulp dietary fiber, 60 6-week-old transgenic rats (30 males, weighing 165–200 g; 30 females, weighing 120–160 g at the commencement of the study) were divided into twelve treatment groups (*n* = 5 per group). Animals in each group received a single oral administration of the test substance via gavage under controlled laboratory conditions. Male and female Wistar rats were randomly allocated to one of six experimental groups: a vehicle control group (10 mL/kg propylene glycol, *n* = 5 per sex), three dose-response treatment groups receiving cassava pulp fiber at 250, 500, and 1000 mg/10 mL/kg body weight (*n* = 5 per sex per group), and two satellite groups (vehicle control and 1000 mg/10 mL/kg cassava pulp fiber, *n* = 5 per sex per group) designed for the assessment of potential delayed toxicological effects.

### 2.7. Preparation of Animals (Sub-Acute Toxicity)

Following a 7-day acclimatization, experimental animals received daily oral administrations of test substances for 28 consecutive days, during which clinical signs of toxicity and mortality were recorded for LD_50_ determination. Animals experiencing severe pain or distress were euthanized, and post-mortem samples were collected. Daily food intake and weekly body weight were monitored. Satellite groups (5 and 6) were observed for an additional 14-day, post-28-day treatment, followed by sample collection consistent with groups 1–4.

### 2.8. Hematological and Biochemical Analysis

The hematological parameters examined were CBC (hematocrit, red blood cell count (RBC), white blood cell count (WBC), hemoglobin, mean corpuscular volume (MCV), mean corpuscular hemoglobin (MCH), mean corpuscular hemoglobin (MCHC), lymphocytes, and platelets), alkaline phosphatase (ALP), alanine aminotransferase (ALT), aspartate aminotransferase (AST), blood urea nitrogen (BUN), creatinine, glucose, triglycerides (TG), total cholesterol (TC), sodium, potassium, total protein, and albumin.

### 2.9. Histopathological Analysis

Necropsy was then performed to determine the gross lesions of various visceral organs. The lungs, hearts, livers, spleens, kidneys, adrenal glands, testis, or ovaries were weighed, and the organ weights were calculated according to relative organ weights (g/100 g body weight). The visceral organs were then preserved in 10% neutral buffer formalin and subsequently subjected to a histological process for preparing tissue slides stained with hematoxylin and eosin (H&E) stains. Tissue slides were histopathologically examined by a veterinary pathologist.

Upon completion of the experimental period and subsequent euthanasia, selected organs from all rats were meticulously excised and promptly preserved in 10% neutral buffered formalin. Following adequate fixation, tissue samples underwent standard histological processing, including dehydration, clearing, and paraffin embedding, in preparation for microscopic examination. Briefly, dehydration was achieved through sequential immersion in a graded ethanol series (50%, 60%, 70%, 80%, 95%, and absolute ethanol [*v*/*v*]), followed by clearing in xylene. The cleared tissues were then infiltrated with molten paraffin wax and embedded in embedding cassettes. The paraffin-embedded tissues were sectioned at a thickness of approximately 4 µm using a rotary microtome. The sections were floated on a water bath maintained at 50 °C, mounted onto glass microscope slides, and dried in a hot air oven at 70 °C. Deparaffinization was performed by immersing the sections in xylene, followed by rehydration through a descending ethanol series (100% and 95%) and rinsing in tap water. Histological staining was conducted using hematoxylin and eosin (H&E). The sections were stained with hematoxylin, rinsed in tap water, and counterstained with eosin. After staining, the sections were dehydrated through graded ethanol (95% and 100%), cleared in xylene, and mounted with a permanent mounting medium. The prepared slides were examined under a light microscope for histopathological assessment.

### 2.10. Statistical Analysis

The data presented were expressed as means ± standard error (SE). The data were analyzed for differences between means using one-way analysis of variance (ANOVA). Duncan’s multiple-range test was used as a post hoc comparison for statistical significance (*p* < 0.05). All statistical analyses were performed using SPSS Windows version 13.0 (SPSS Inc., Chicago, IL, USA).

## 3. Results

### 3.1. Acute Toxicity

**Figure 1:**
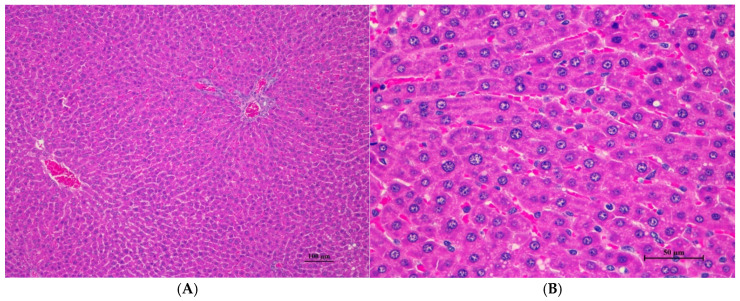
Histopathological examination of rat liver tissue in acute toxicity from a single oral dose of CDF demonstrated minimal and non-pathological changes. (**A**) The hepatic parenchyma showed a normal central vein, hepatic portal triad, and hepatic cord (100×, H&E). (**B**) High magnification shows polygonal hepatocytes with central nuclei. Very mild cytoplasmic swelling and fine vacuolation were observed in some hepatocytes (400×, H&E).

**Figure 2:**
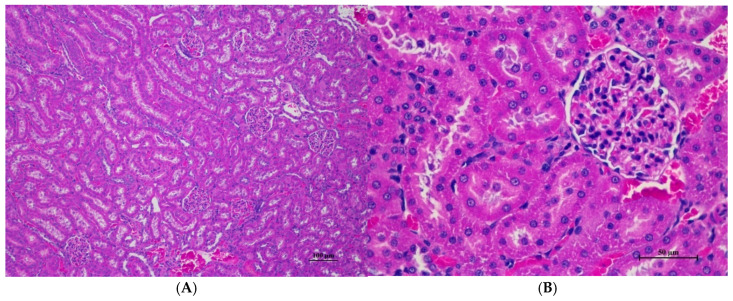
Histopathological examination of rat renal tissue in acute toxicity from a single oral dose of CDF revealed well-preserved histological structure. (**A**) The renal tissue showed a normal histologic appearance with intact glomeruli and tubular structure (100×, H&E). (**B**) High magnification displayed uniform renal tubule with normal glomeruli. Mild cytoplasmic change with no evidence of inflammation, cell death, or necrosis observed (400×, H&E).

**Figure 3:**
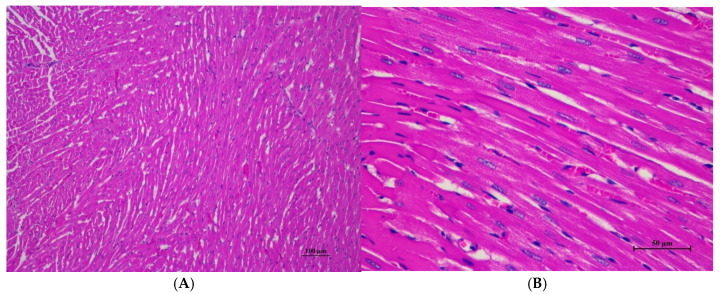
Histopathological examination of rat cardiac tissue in acute toxicity from single oral dose of CDF showed intact histological structure. (**A**) Cardiac muscle fibers are arranged in normal pattern with well histological structure (100×, H&E). (**B**) High magnification showed myocardial with well-defined striations and intact nuclei. Occasional cardiac myocytes exhibited small focal cytoplasmic vacuolation, with no evidence of cell degeneration, necrosis, or inflammatory reaction (400×, H&E).

**Figure 4:**
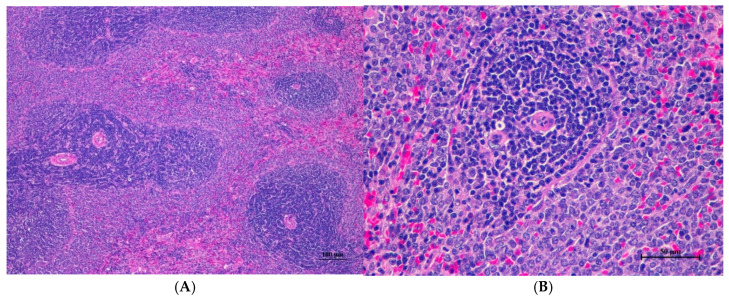
Histopathological examination of rat splenic tissue in acute toxicity from single oral dose of CDF displayed normal microscopic structure. (**A**) Splenic tissue showed intact structure with distinct white pulp and red pulp regions (100×, H&E). (**B**) High magnification reveals normal lymphoid follicles with intact germinal centers. No histopathological abnormalities were observed (400×, H&E).

**Figure 5:**
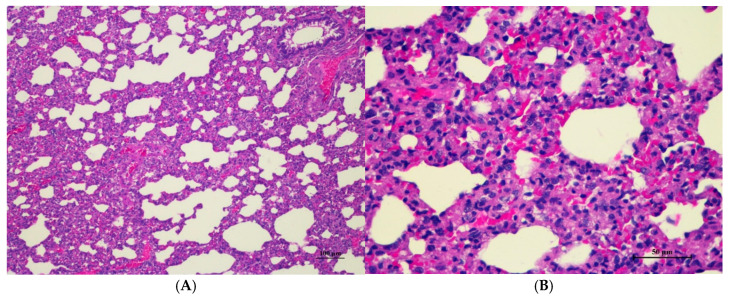
Histopathological examination of rat lung tissue in acute toxicity from a single oral dose of CDF exhibited histopathological changes. (**A**) Lung tissue shows marked alveolar septa thickening with disrupted alveolar structure. Interstitial areas are expanded by cellular infiltrates (100×, H&E). (**B**) High magnification showed septal infiltration by mononuclear cells, hyperemia, and mild hemorrhage in alveolar space (400×, H&E).

The male rats were orally given a single dose of CDF at doses of 175 (175AM), 550 (550AM), and 2000 (2000AM) mg/10 mL/kg, and female rats were given CDF at doses of 175 (175AF), 550 (550AF), and 2000 (2000AF) mg/10 mL/kg, respectively. There were no significant signs of mortality, general behavior, or gross differences in appearance in the internal organs during the 14 days of the testing period. However, there were increases in body weight gain in both the control and the treated groups. A gross and histopathological examination of the liver showed that the internal organs did not reveal any pathological abnormality relative to the control (Table 1, Table 2, Table 3, Table 4, Table 5, Table 6, Table 7, Table 8 and Table 9). It was further confirmed that the CDF did not cause any tissue damage (Figure 1, Figure 2, Figure 3, Figure 4 and Figure 5). 

In male rats, a significant increase in average body weight was observed in all treatment groups at weeks 1 and 2 compared to week 0 (*p* < 0.05). Additionally, male rats exhibited a significant increase in average body weight at week 2 compared to week 1 (*p* < 0.05). However, no significant differences in average body weight were found among treatment groups within either week 1 or week 2 (*p* > 0.05). Similarly, female rats showed a significant increase in average body weight in all treatment groups at weeks 1 and 2 compared to week 0 (*p* < 0.05), with no significant differences observed among groups within either week (*p* > 0.05).

#### 3.1.1. Body Weight Gain of Male and Female Rats

Table 2 presents the mean body weight gain of male and female rats administered cassava pulp dietary fiber at doses of 175, 550, and 2000 mg/kg (10 mL/kg volume). In male rats, a significant increase in mean body weight was observed in all treatment groups at week 2 compared to week 0 (*p* < 0.05). No significant differences were found in weight gain between weeks 1 and 2 within the same treatment groups. Similarly, female rats exhibited a significant increase in mean body weight at week 2 compared to week 0 (*p* < 0.05) across all doses. No significant differences were observed in weight gain between groups at week 1 (*p* > 0.05). However, at week 2, the 175 mg/kg female group showed a significantly higher mean body weight compared to the control (*p* < 0.05).

#### 3.1.2. Food Consumption of Male and Female Rats

Table 3 presents the mean food consumption of male and female rats administered cassava pulp dietary fiber at 175, 550, and 2000 mg/10 mL/kg. In males, food intake significantly increased in all treatment groups at week 2 compared to week 0 (*p* < 0.05). Notably, the 2000 mg/10 mL/kg group exhibited higher food intake than the 550 mg/10 mL/kg group at week 2 (*p* < 0.05). In females, a similar increase in food intake at week 2 compared to week 0 was observed across all treatment groups (*p* < 0.05). Additionally, the 175 mg/10 mL/kg female group showed significantly higher food intake than the control at week 2 (*p* < 0.05). No significant differences were found in food intake among treatment groups within week 1 for either sex (*p* > 0.05).

#### 3.1.3. Relative Organ Weight of Male and Female Rats

Relative organ weights (ROWs) in male and female rats following sub-acute dietary fiber administration are presented in Table 4 and Table 5. In males, the 175 mg/kg dose (175AM) resulted in a decreased relative liver weight compared to the control and 550 mg/kg (550AM) groups. Notably, the 550AM and 2000 mg/kg (2000AM) groups exhibited significantly increased relative seminal vesicle and coagulating gland weights compared to the 175AM group (*p* < 0.05). No significant differences were observed in the relative weights of kidneys, heart, spleen, lung, adrenal gland, thymus gland, brain, testes, and epididymis (*p* > 0.05). In females (Table 5), the 550 mg/kg dose (550AF) significantly reduced relative brain weight compared to the control (*p* < 0.05). Both the 175 mg/kg (175AF) and 550AF groups showed a significant decrease in relative uterus weight compared to the control (*p* < 0.05). No significant differences were found in the relative weights of kidneys, heart, spleen, lung, adrenal gland, thymus gland, and ovaries (*p* > 0.05).

#### 3.1.4. Biochemical Parameters of Male and Female Rats

Table 6 and Table 7 present the biochemical parameters of male and female rats administered cassava pulp dietary fiber at 175, 550, and 2000 mg/kg (AM for males, AF for females). In males (Table 6), triglyceride levels were reduced in the 175AM and 550AM groups compared to controls. In females (Table 7), the 550AF group exhibited a significant decrease in ALP (*p* < 0.05). Furthermore, the 550AF group showed a reduction in AST, while the 2000AF group displayed a significant decrease in ALT (*p* < 0.05). Across all treated groups, potassium levels were significantly lower than controls (*p* < 0.05). Sodium levels were reduced in the 550AF and 2000AF groups, with the 550AF group demonstrating a significantly lower level than the 175AF group (*p* < 0.05).

#### 3.1.5. Hematological Parameters of Male and Female Rats

Table 8 and Table 9 detail the hematological parameters of male and female rats administered cassava pulp dietary fiber at doses of 175, 550, and 2000 mg/10 mL/kg. In male rats (Table 8), no significant differences were observed across all hematological parameters evaluated (*p* > 0.05). However, in female rats (Table 9), the 550 mg/10 mL/kg group exhibited significantly elevated platelet counts compared to the 175 mg/10 mL/kg group (*p* < 0.05). All other hematological parameters in female rats remained unaffected by treatment.

#### 3.1.6. Signs of Toxicity and Mortality in Male and Female Rats

In both male and female rats, CDF was administered at doses of 175, 550, and 2000 mg/10 mL/kg (designated as 175AM/AF, 550AM/AF, and 2000AM/AF, respectively). No signs of toxicity or mortality were observed in any treatment group, including assessments of dermal, gastrointestinal, respiratory, and locomotor functions (Table 10 and Table 11).

#### 3.1.7. Histopathological of Male and Female Rats

Histopathological findings for male and female rats administered CDF at 175, 550, and 2000 mg/10 mL/kg are summarized in Table 12 and Table 13, respectively. In male rats (Table 12), the 2000 mg/kg group exhibited interstitial pneumonitis (IP) in the lungs of four out of five animals. The 550 mg/kg group showed mild hepatic vacuolation (HV) and hepatic degeneration (HD) in two out of five animals as well as IP in the lungs (four out of five). The 175 mg/kg group presented with mild HV and HD in the liver (one of five) and IP or normal alveolar septal thickness (N AVT) in the lungs (two of five each). The propylene glycol control group displayed mild HV and HD in the liver (two of five) and IP or N AVT in the lungs (three of five and one of five, respectively). In female rats (Table 13), all doses, including the control, resulted in IP in the lungs of the majority or all animals. The 550 mg/kg group also showed mild HV and HD in the liver (one of five). No histopathological alterations were observed in the kidneys, hearts, or spleens of either sex across all treatment groups.

Histopathological analysis revealed generally unremarkable findings in the liver, kidney, heart, and spleen, with only minor, scattered hepatocellular swelling and vacuolation in the liver, mild renal tubular degeneration in limited kidney areas, and focal myocardial vacuolation and lymphoid aggregation in the heart. Conversely, the lung exhibited significant pathological changes, characterized by alveolar septal thickening due to severe interstitial infiltration of lymphocytes and other mononuclear cells, nodular lymphoid aggregations, alveolar septal engorgement and hyperemia, alveolar edema, and multifocal mild alveolar hemorrhage, suggesting a potential site of inflammatory response.

Histopathologies of the liver, kidney, heart, spleen, and lung are shown in Figure 1, Figure 2, Figure 3, Figure 4 and Figure 5.

### 3.2. Sub-Acute Toxicity

#### 3.2.1. Effect on Body Weight of Male and Female Rats

The effects of cassava pulp dietary fiber on rat body weight were evaluated over a four-week sub-acute toxicity study. Male and female rats were administered the fiber at doses of 250, 500, and 1000 mg/kg (designated as 250RM/RF, 500RM/RF, and 1000RM/RF, respectively). Table 14 and Table 15 detail the weekly average body weight changes for males and females, respectively. In both sexes, all treated groups and the control exhibited significant increases in body weight (*p* < 0.05) from week 1 to weeks 2, 3, and 4. Within each week, no significant differences in average body weight were observed between the treated groups and the control. The satellite groups, monitored for an additional two weeks, also showed significant body weight increases (*p* < 0.05) from week 1 to weeks 3–6, with no inter-group differences within each week. These results indicate that cassava pulp dietary fiber, at the tested doses, did not negatively impact body weight gain in either male or female rats during the sub-acute study.

#### 3.2.2. Weight Gain of Male and Female Rats for 28 Days

Table 16 and Table 17 present the mean body weight changes in male and female rats, respectively, following daily oral administration of cassava pulp dietary fiber at doses of 250, 500, and 1000 mg/kg. Both male and female rats in all treatment groups, including the control, exhibited significant increases in mean body weight from week 2 to week 4 compared to week 1 (*p* < 0.05). Similarly, in the satellite groups, significant weight gain (*p* < 0.05) was observed from week 3 to week 6 relative to week 1 (*p* < 0.05). Within each week, no statistically significant differences in mean body weight were observed across treatment groups for either sex (*p* > 0.05). All groups demonstrated a consistent increase in mean body weight throughout the experimental period.

#### 3.2.3. Food Consumption of Male and Female Rats for 28 Days

Table 18 and Table 19 present the mean food consumption of male and female rats administered cassava pulp dietary fiber at 250, 500, and 1000 mg/kg. For male rats (Table 18), week 1 showed a significant decrease in food intake at 250 and 1000 mg/kg compared to controls (*p* < 0.05). Conversely, weeks 2–4 demonstrated significantly increased food intake in all treated groups relative to week 1 (*p* < 0.05). In the satellite study, the 1000 mg/kg group showed a decreased intake in week 1, followed by a progressive increase in weeks 3–6. For female rats (Table 19), consistent significant decreases in food intake were observed at 500 and 1000 mg/kg compared to controls across weeks 1–4 (*p* < 0.05). The satellite study showed a progressive increase in food intake in weeks 4–6, with no significant differences between treated and control groups within each week.

#### 3.2.4. Relative Organ Weight of Male and Female Rats

Table 20 and Table 21 detail the relative organ weights of rats administered cassava pulp dietary fiber. In male rats (Table 20), the 1000 mg/kg dose significantly increased liver weight compared to controls and the 500 mg/kg group, with satellite data confirming increased liver and seminal vesicle/coagulating gland weights at this dose; other organs showed no significant changes. Conversely, in female rats (Table 21), the 500 and 1000 mg/kg doses resulted in decreased liver and kidney weights, and the 1000 mg/kg dose also reduced heart weight, relative to controls and the 250 mg/kg group; no significant differences were observed in other female organ weights, including in the satellite study (*p* > 0.05).

#### 3.2.5. Biochemical Parameters of Male and Female Rats for 28 Days

Table 22 and Table 23 summarize the biochemical parameters of male and female rats, respectively, administered cassava pulp dietary fiber at 250, 500, and 1000 mg/kg. No significant differences were observed in any measured biochemical parameters (blood glucose, BUN, creatinine, TC, TG, total protein, albumin, AST, ALT, APT, potassium, and sodium) between treated and control groups in either male or female rats, including satellite studies (*p* > 0.05).

#### 3.2.6. Hematological Parameters of Male and Female Rats for 28 Days

Table 24 and Table 25 summarize hematological parameters in male and female rats administered cassava pulp dietary fiber at 250, 500, and 1000 mg/kg. No significant differences were observed between treated, satellite, and control groups for any measured hematological parameters, including white blood cell counts, differential leukocyte counts, red blood cell indices, and platelet counts, in either sex (*p* > 0.05).

#### 3.2.7. Signs of Toxicity and Mortality in Male and Female Rats

The 28-day administration of cassava pulp dietary fiber at 250, 500, and 1000 mg/kg to male and female rats (Table 26 and Table 27) did not induce any observable signs of toxicity or mortality. Specifically, no abnormalities were noted in skin, digestive, respiratory, or motor functions across all treated and satellite (1000 mg/kg) groups.

#### 3.2.8. Histopathological of Male and Female Rats for 28 Days

Table 28 and Table 29 detail histopathological findings in male and female rats, respectively, following cassava pulp dietary fiber administration at 250, 500, and 1000 mg/kg.

For male rats (Table 28), all treated groups, including the control, exhibited mild hepatic vacuolation (HV) and hepatic degeneration (HD) in the liver. The 1000 mg/kg and 500 mg/kg groups showed HV/HD in four out of five cases, the 250 mg/kg group in five out of five cases, and the control in three out of five cases. Interstitial pneumonitis (IP) was observed in the lungs of all groups, with varying incidence. The satellite 1000 mg/kg group showed HV/HD in the liver (one of five) and IP, normal with alveolar septal thickness (N AVT), and normal with alveolar hyperemia (N AVHy) in the lungs. No histopathological changes were observed in kidneys, hearts, or spleens.

**Figure 6:**
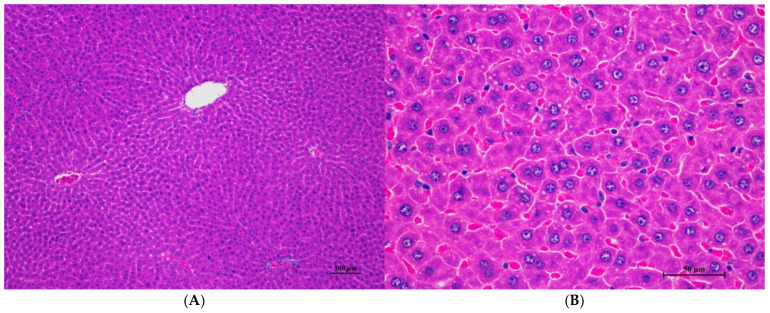
Histopathological examination of rat liver tissue in sub-acute toxicity from daily (28 days) fed oral dose of CDF revealed infrequent findings and no prominent lesions. (**A**) The liver tissue showed an intact lobular arrangement of the hepatic cord and central vein (100×, H&E). (**B**) High magnification showed structurally normal hepatocytes with distinct cellular boundaries and centrally located nuclei. Occasional hepatocytes contained fine cytoplasmic vacuolation (400×, H&E).

**Figure 7:**
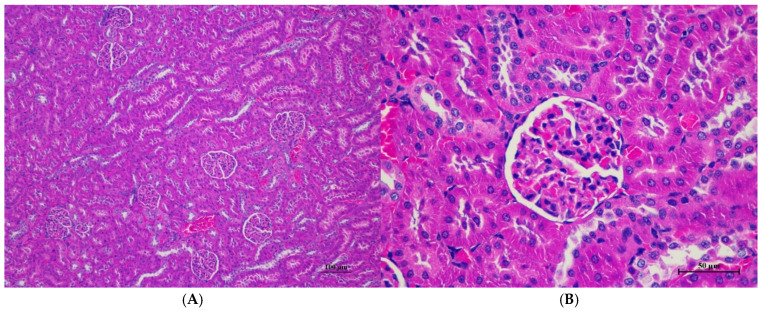
Histopathological examination of rat kidney tissue in sub-acute toxicity from daily (28 days) fed oral dose of CDF revealed normal histological structure. (**A**) The renal tissue exhibited intact glomeruli and well-organized renal tubules (100×, H&E). (**B**) High magnification showed normal size and cellular composition of glomeruli. The surrounding renal tubules demonstrated well epithelial lining with intact cellular morphology (400×, H&E).

**Figure 8:**
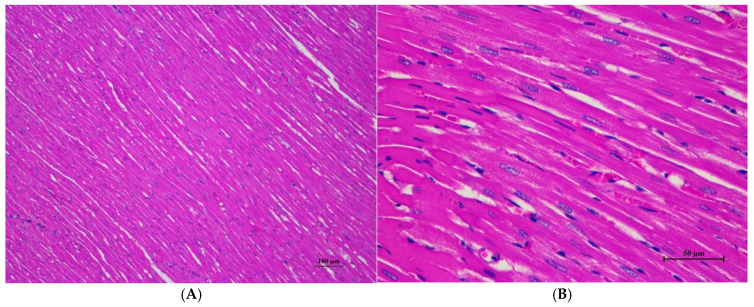
Histopathological examination of rat cardiac tissue in sub-acute toxicity from daily (28 days) fed oral dose of CDF showed intact histological structure. (**A**) Myocardial tissue exhibited normal morphology and arrangement of cardiomyocytes (100×, H&E). (**B**) High magnification demonstrated cardiomyocytes with clear cross-striations, intact nuclei, and cytoplasmic structure (400×, H&E).

**Figure 9:**
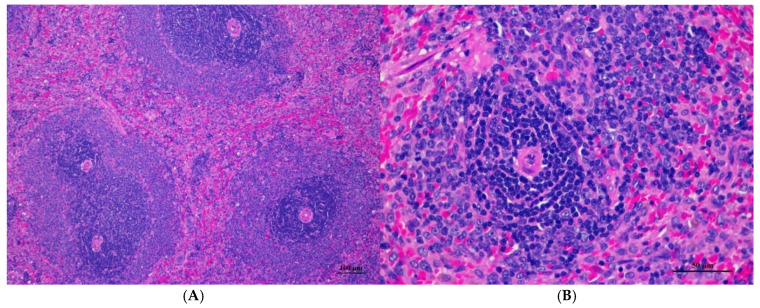
Histopathological examination of rat splenic tissue in sub-acute toxicity from daily (28 days) fed oral dose of CDF showed normal microscopic appearances. (**A**) Splenic tissue exhibited well-organized white pulp, marginal zone, and red pulp (100×, H&E). (**B**) High magnification showed an unremarkable lesion of red pulp. The lymphoid follicle displayed an intact germinal center with densely packed lymphocytes (400×, H&E).

**Figure 10:**
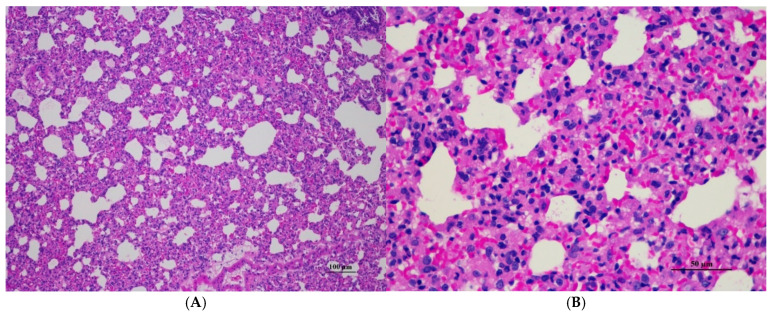
Histopathological examination of rat lung tissue in sub-acute toxicity from daily (28 days) fed oral dose of CDF showed histopathological alterations. (**A**) Pulmonary parenchyma reveals prominent thickening of alveolar septa with infiltration of inflammatory cells (100×, H&E). (**B**) High magnification showed alveolar septa infiltrated by numerous mononuclear cells. Capillary engorgement and focal hemorrhage were observed (400×, H&E).

Table 29 details histopathological findings in female rats following cassava pulp dietary fiber administration. Liver changes, characterized by mild hepatic vacuolation (HV) and hepatic degeneration (HD), were observed across all treated groups and controls, including satellite groups, with varying incidence. Lung findings included interstitial pneumonitis (IP) and alveolar septal thickening (AVT) in treated and control groups. Specifically, the 1000 mg/kg group showed HV/HD in all subjects and IP in four out of five, including the satellite group. The 500 mg/kg group showed HV/HD in all subjects and IP in one out of five. The 250 mg/kg group showed HV/HD in four out of five and diffused HV in one out of five, with IP in four out of five. Controls also exhibited HV/HD and IP. No histopathological changes were observed in the kidneys, heart, or spleen in any group. Representative histopathological images are presented in Figure 6, Figure 7, Figure 8, Figure 9 and Figure 10.

Histopathological analysis of rat organs following 28-day cassava fiber administration revealed mild hepatocellular swelling and vacuolation in the liver, deemed within normal limits, while the kidneys, heart, and spleen exhibited no significant lesions; however, the lungs consistently displayed moderate to severe alveolar edema, septal engorgement, hemorrhage, and inflammatory cell infiltration, suggesting a potential pulmonary response to the cassava fiber, despite the absence of overt clinical toxicity.

## 4. Discussion

This study evaluated the acute and sub-chronic (28-day) toxicity of CDF in Wistar rats. In the acute toxicity assessment, oral administration of CDF at the highest tested dose of 2000 mg/kg body weight in both male and female rats did not elicit any observable adverse toxicological effects, indicating the LD_50_ was higher than this dose. Similarly, in the 28-day sub-acute toxicity study, daily oral administration of CDF at the maximum tested dose of 1000 mg/kg body weight in both sexes did not induce any signs of toxicity or adverse effects, identifying the NOAEL for sub-acute exposure at 1000 mg/kg body weight.

### 4.1. Acute Toxicity Study

The assessment of CDF’s influence on body weight, body weight gain, and average food intake revealed that, during the second week following a single oral administration of CDF, both male and female rats exhibited a statistically significant increase in body weight and body weight gain compared to baseline measurements (week 0). Specifically, the 2000 mg/kg CDF-treated male group demonstrated a significantly higher average food intake than the 550 mg/kg CDF-treated male group. In female rats, the 175 mg/kg CDF-treated group showed a statistically significant increase in body weight but not food intake relative to the control group.

Male rats had no effect on the relative organ weights of the kidneys, heart, spleen, lungs, adrenals, thymus, brain, testes, and epididymis. In the 175AM group, the weight of the liver decreased more than in the control group and 550AM group. In addition, the seminal vesicle and coagulating gland weights were lower in the 175AM group than in the 550AM and 2000AM groups. In female rats, the relative weights of the liver, kidneys, heart, spleen, lungs, adrenal glands, thymus, and ovaries were unaffected. However, the 550AF group showed decreased brain weight compared to the control group, and both the 550AF and 175AF groups had lower uterine weights than the control group. There was little change in the increase or decrease in the organ weight of the rats. This change may be due to differences in the size of the animals’ internal organs [7].

In male rats, the 175AM and 550AM groups demonstrated significantly reduced triglyceride levels, and the 550AM group showed a significant decrease in alkaline phosphatase levels compared to the control group. For female rats, the 550AF group had significantly lower aspartate aminotransferase (AST) levels, the 2000AF group had significantly lower alanine aminotransferase (ALT) levels, and potassium levels were significantly decreased in the 175AF, 550AF, and 2000AF groups. Sodium levels were also significantly lower in the 550AF and 2000AF female rat groups compared to the control. All other measured biochemical parameters in both sexes were comparable to the control group. Importantly, the observed alterations in biochemical values were minimal and remained within the normal physiological range [6]. Furthermore, the treatment did not significantly impact hematological parameters in either sex.

Histopathological changes in the liver of male rats including hepatic vacuolation and hepatic degeneration were found in the control group and 175AM, 550AM, and 550AF groups. Abnormalities were found in the cytoplasm of liver cells, to a mild degree and diffuse in some areas of the liver, and portal tracts were normal. These findings were minimal and considered non-significant lesions. Furthermore, biochemical values were minimally changed and remained within the normal range, which led to the interpretation of the organ being normal.

Lung abnormalities in the lung tissue of both sexes were found, i.e., interstitial pneumonitis. The histopathologic features of the lungs show varying severity degrees of alveolar edema, alveolar septal engorgement, alveolar hemorrhage, and inflammatory cell infiltration to pulmonary parenchyma. In all groups, this abnormality may indicate infection from laboratory animals. However, further investigation is required. Cassava pulp dietary fiber was found to be well tolerated in a short-term toxicity assessment with non-toxic thresholds of 10.01 g/kg body weight/day for male rats and 11.21 g/kg body weight/day for female rats. Cassava pulp dietary fiber also showed cholesterol-lowering effects [4].

Cellulose derivatives, including mechanically fibrillated cellulose nanofibers (fib-CNF), sodium carboxymethyl cellulose (Na-CMC), and methylcellulose (MC), exhibit low acute oral toxicity in rodents. Studies following OECD guidelines and LD_50_ estimations demonstrate high tolerance, with no significant mortality or severe toxicological effects observed at doses up to 5000 mg/kg/day. Overall, these results indicate practical non-toxicity for cellulose-based material [7,8,9].

### 4.2. Sub-Acute Toxicity Study

After 28 days, both male and female rats had increases in body weight and body weight gain and food intake when compared with week 0 in all groups, and no difference from the control group was found. Week 6, in the satellite group (1000 mg/10 mL/kg), showed an increase in body weight, body weight gain, and food intake when compared to week 0 in all groups. There was no difference from the control satellite group.

Male rats showed no effect on the relative organ weights of the kidney, heart, spleen, lung, adrenal gland, thymus, brain, testes, and epididymis. The 1000RM group showed a higher liver weight than the control group and the 500RM group. In the satellite group, the 1000RM group had an increased liver weight and seminal vesicles/coagulating glands than the control satellite group. Female rats, in all groups, had no effect on the relative organ weights of the spleen, lung, adrenal gland, thymus, brain, and ovaries and uterus. 500RF and 1000RF groups showed a lower liver weight than the control group. 500RF and 1000RF groups showed a lower kidney weight than the control group and the 250RF group. The 1000RF group showed a decreased heart weight compared to the 250RF group. There was little change in the increase or decrease in the organ weight of the rats. This change may be due to differences in the size of the animals’ internal organs [7].

Biochemistry and hematology parameters in rats of both sexes were not different between the control group and between the experimental groups.

Rats of both sexes, with respect to histopathological changes in the liver, kidney, heart, and spleen, were normal. Histopathological changes in the liver, including hepatic vacuolation and hepatic degeneration, were found in all groups of both sexes. These changes were found to be abnormal in the cytoplasm of liver cells, to a mild degree and diffuse in some areas of the liver, and portal tracts were normal. These findings represent only minimal and non-significant pathologic changes. Additionally, biochemical values were within normal limits, indicating this organ remained normal.

Abnormalities were found in the lung tissue of both sexes, i.e., interstitial pneumonitis. The histopathologic feature of the lungs shows varying severity degrees of alveolar edema, alveolar septal engorgement, alveolar hemorrhage, and inflammatory cell infiltration to pulmonary parenchyma in all groups. Normal lung tissue, except for alveolar septal thickness and alveolar hyperemia, was found in both satellite groups. This abnormality may indicate infection from the facility used for laboratory animals. However, further investigation is required.

The National Laboratory Animal Center [9] reported that rats of 4–7 weeks of age have a red blood count of approximately 5.7–8.6 (×10^6^ cell/mm^3^), white blood cell count of approximately 5.7–8.6 (×10^3^ cell/mm^3^), hemoglobin concentration of approximately 12.3–15.9 g/dL, and MCV, MCH, and MCHC in the range of approximately 51.3–64.1 ×m^3^/red cell, 18.6–21.5 pg/red cells, and 33.5–36.2 g/dL, respectively. So, the standard values obtained from the National Laboratory Animal Center support those of this study [6].

Based on the reviewed sub-chronic toxicity studies, the observation of increased body weight, body weight gain, and food intake compared to baseline within the treatment groups, without a significant difference from the control group, is a finding that is resounded in some studies of other plant-derived fibers like NawaTab, pumpkin pectin, guar gum, and Fitnox. However, some fibers, like cellulose nanofibers, showed a decrease in body weight in the recovery period, and one study on cassava pulp fiber in hypercholesterolemic rats showed an increase compared to the control. This suggests that the effects on body weight and food intake in sub-chronic toxicity studies can vary depending on the specific type of fiber, the dosage, the duration of the study, and the physiological state of the animals. This finding of increases within the treated groups that are not significantly different from the control group indicates a general trend of no major adverse impact on these parameters for the tested fiber under the specified conditions [10,11,12,13,14,15,16].

These results suggest a promising avenue for the incorporation of dietary fiber prepared from cassava pulp as a novel food ingredient in functional food formulations. Moving forward, it is crucial to acknowledge the regulatory landscape, particularly the stipulations outlined by the Thailand Federal Drug Administration, Ministry of Public Health in Thailand, which mandates rigorous toxicity assessments for all new food ingredients prior to commercial application [17].

## 5. Conclusions

In summary, this study established the acute and 28-day sub-acute oral toxicity profiles of dietary fiber extracted from cassava pulp in Wistar rats. The LD_50_ was higher than 2000 mg/kg for single doses, and the NOAELs were determined to be 2000 mg/kg for acute exposure and 1000 mg/kg for repeated daily dosing, indicating a high margin of safety. These findings support the potential application of cassava pulp fiber in future in vivo and human studies, emphasizing its promising safety profile. However, the observed pulmonary histopathological abnormalities across all groups, including controls, necessitate further investigation to determine their etiology and prevent recurrence. While potential environmental factors, such as barn-related infections, are considered, a comprehensive assessment is crucial to ensure the reliability and translatability of these results.

## Figures and Tables

**Table 1:** Body weight of male rats in acute toxicity study.

Group	Male			Female		
	Body Weight (g)			Body Weight (g)		
	Week 0	Week 1	Week 2	Week 0	Week 1	Week 2
Control	214 ± 5.42	265 ± 9.84 ^m^	330 ± 13.35 ^mn^	147 ± 2.85	172 ± 5.18 ^m^	193 ± 4.18 ^mn^
175	209 ± 5.42	252 ± 6.75 ^m^	315 ± 9.84 ^mn^	153 ± 8.40	187 ± 9.62 ^m^	215 ± 14.03 ^mn^
550	203 ± 4.18	261 ± 9.42 ^m^	317 ± 13.65 ^mn^	155 ± 3.95	184 ± 3.71 ^m^	209 ± 2.74 ^mn^
2000	213 ± 11.54	263 ± 9.94 ^m^	328 ± 9.94 ^mn^	152 ± 3.79	180 ± 5.86 ^m^	207 ± 6.27 ^mn^

Values expressed as mean ± S.E.M. (*p* < 0.05; two-way ANOVA; Tukey Method). ^m^ Significantly different from week 0, ^n^ significantly different from week 1.

**Table 2:** Weight gain of male rats in acute toxicity study.

Group	Male		Female	
	Weight Grain from Week 0 (g)		Weight Grain from Week 0 (g)	
	Week 1	Week 2	Week 1	Week 2
Control	51 ± 4.81	116 ± 9.91 ^n^	25 ± 3.06	46 ± 2.09 ^n^
175AM	43 ± 2.24	106 ± 4.81 ^n^	34 ± 4.81	62 ± 6.52 ^an^
550AM	58 ± 12.32	114 ± 16.81 ^n^	29 ± 1.12	54 ± 2.09 ^n^
2000AM	50 ± 6.85	115 ± 6.85 ^n^	28 ± 2.85	55 ± 3.95 ^n^

Values expressed as mean ± S.E.M. (*p* < 0.05; two-way ANOVA; Tukey Method). ^a^ Significantly different from 550AM, ^n^ Significant difference from week 1.

**Table 3:** Food consumption of male and female rats in acute toxicity study.

Group	Male		Female	
	Food Intake from Week 0 (g)		Food Intake from Week 0 (g)	
	Week 1	Week 2	Week 1	Week 2
Control	23.21 ± 0.49	24.50 ± 0.79 ^n^	15.47 ± 0.43	24.50 ± 0.79 ^n^
175AM	22.18 ± 0.11	23.62 ± 0.12 ^n^	15.79 ± 0.64	23.62 ± 0.12 ^n^
550AM	22.30 ± 0.15	23.12 ± 0.28 ^n^	16.28 ± 0.50	23.12 ± 0.28 ^n^
2000AM	22.07 ± 1.44	26.19 ± 1.02 ^cn^	15.61 ± 0.64	26.19 ± 1.02 ^cn^

Values expressed as mean ± S.E.M. (*p* < 0.05; two-way ANOVA; Tukey Method). ^c^ Significantly different from 550AM, ^n^ significantly different from week 1.

**Table 4:** Relative organ weight of male rats in acute toxicity study.

Group	ROW (g per 100 g Body Weight)										
	Liver	Kidneys	Heart	Spleen	Lung	Adrenal	Thymus	Brain	Testes	Epididymis	Seminal Vesicles
						Gland	Gland				Coagulating Glands
Control	4.64 ± 0.97	0.90 ± 0.12	0.33 ± 0.07	0.31 ± 0.26	0.66 ± 0.51	0.03 ± 0.01	0.02 ± 0.02	0.63 ± 0.06	0.99 ± 0.43	0.24 ± 0.14	0.20 ± 0.05
175AM	3.78 ± 0.61 ^a^	0.83 ± 0.12	0.32 ± 0.05	0.20 ± 0.04	0.61 ± 0.30	0.02 ± 0.01	0.01 ± 0.01	0.66 ± 0.02	1.08 ± 0.09	0.25 ± 0.05	0.19 ± 0.04
550AM	4.73 ± 0.91 ^b^	0.91 ± 0.09	0.31 ± 0.04	0.24 ± 0.02	0.48 ± 0.07	0.03 ± 0.01	0.01 ± 0.01	0.62 ± 0.04	1.09 ± 0.16	0.27 ± 0.13	0.26 ± 0.08 ^b^
2000AM	4.36 ± 1.18	0.85 ± 0.12	0.30 ± 0.04	0.24 ± 0.04	0.44 ± 0.03	0.02 ± 0.01	0.01 ± 0.02	0.63 ± 0.03	1.02 ± 0.19	0.29 ± 0.06	0.27 ± 0.06 ^b^

Values expressed as mean ± S.E.M. (*p* < 0.05; one-way ANOVA). ^a^ Significantly different from control, ^b^ significantly different from 175AM. Relative weight of organ: ROW.

**Table 5:** Relative organ weight of female rats in acute toxicity study.

Group	ROW (g per 100 g Body Weight)									
	Liver	Kidneys	Heart	Spleen	Lung	Adrenal	Thymus	Brain	Ovary	Uterus
						Gland	Gland			
Control	3.74 ± 0.49	0.86 ± 0.08	0.28 ± 0.08	0.26 ± 0.01	0.58 ± 0.08	0.05 ± 0.01	0.02 ± 0.01	0.97 ± 0.01	0.11 ± 0.01	0.28 ± 0.06
175AF	3.71 ± 0.56	0.84 ± 0.09	0.30 ± 0.03	0.22 ± 0.03	0.54 ± 0.03	0.04 ± 0.01	0.01 ± 0.00	0.89 ± 0.04	0.09 ± 0.02	0.20 ± 0.02 ^a^
550AF	3.66 ± 0.38	0.86 ± 0.04	0.31 ± 0.02	0.25 ± 0.03	0.56 ± 0.06	0.04 ± 0.01	0.02 ± 0.01	0.86 ± 0.04 ^a^	0.10 ± 0.01	0.19 ± 0.02 ^a^
2000AF	3.75 ± 0.70	0.87 ± 0.08	0.31 ± 0.02	0.24 ± 0.02	0.58 ± 0.07	0.04 ± 0.01	0.02 ± 0.01	0.91 ± 0.04	0.09 ± 0.01	0.22 ± 0.02

Values expressed as mean ± S.E.M. (*p* < 0.05; one-way ANOVA). ^a^ Significantly different from control. Relative weight of organ: ROW.

**Table 6:** Biochemical parameters of male rats in acute toxicity study.

Group	Parameters											
	Blood Sugar	BUN	Creatinine	TC	TG	Total Protein	Albumin	AST	ALT	ALP	Potassium	Sodium
	(mg/dL)	(mg/dL)	(mg/dL)	(mg/dL)	(mg/dL)	(g/dL)	(g/dL)	(U/L)	(U/L)	(U/L)	(mmol/L)	(mEq/L)
Control	101.80 ± 9.23	17.40 ± 0.22	0.32 ± 0.05	65.60 ± 5.46	129.00 ± 11.90	6.50 ± 0.60	3.38 ± 0.10	158.67 ± 20.10	36.60 ± 5.89	162.00 ± 11.43	9.20 ± 0.17	186.78 ± 5.68
175AM	112.80 ± 8.00	17.30 ± 0.69	0.41 ± 0.05	54.00 ± 2.72	83.00 ± 7.56 ^a^	5.76 ± 0.03	3.28 ± 0.02	118.00 ± 28.62	39.40 ± 6.61	145.00 ± 7.07	7.97 ± 0.91	173.68 ± 7.34
550AM	89.80 ± 10.16	16.08 ± 1.61	0.40 ± 0.07	64.20 ± 3.63	95.60 ± 14.32 ^a^	6.08 ± 0.13	3.36 ± 0.08	121.75 ± 24.00	33.80 ± 4.23	124.20 ± 5.02 ^a^	7.16 ± 0.13	181.38 ± 10.95
2000AM	105.20 ± 6.46	18.50 ± 1.30	0.36 ± 0.03	60.60 ± 2.17	104.00 ± 15.22	6.04 ± 0.12	3.36 ± 0.08	114.75 ± 14.38	29.00 ± 3.71	134.80 ± 9.79	8.79 ± 0.70	172.50 ± 2.52

Values expressed as mean ± S.E.M. (*p* < 0.05; one-way ANOVA). ^a^ Significantly different from control. BUN: blood urea nitrogen, TC: total cholesterol, TG: triglycerides, AST: aspartate aminotransferase, ALT: alanine aminotransferase, ALP: alkaline phosphatase.

**Table 7:** Biochemical parameters of female rats in acute toxicity study.

Group	Parameters											
	Blood Sugar	BUN	Creatinine	TC	TG	Total Protein	Albumin	AST	ALT	ALP	Potassium	Sodium
	(mg/dL)	(mg/dL)	(mg/dL)	(mg/dL)	(mg/dL)	(g/dL)	(g/dL)	(U/L)	(U/L)	(U/L)	(mmol/L)	(mEq/L)
Control	82.00 ± 3.64	16.16 ± 0.81	0.37 ± 0.04	60.80 ± 6.31	54.80 ± 4.10	60.80 ± 6.31	3.50 ± 0.13	194.66 ± 13.83	38.40 ± 5.87	72.40 ± 4.48	10.15 ± 0.39	167.84 ± 1.63
175AF	85.00 ± 4.62	15.48 ± 1.68	0.48 ± 0.04	79.00 ± 28.25	48.60 ± 5.72	53.75 ± 1.44	3.82 ± 0.14	140.20 ± 19.92	25.20 ± 2.48	93.25 ± 9.40	6.74 ± 0.56 ^a^	166.04 ± 1.91
550AF	76.60 ± 8.70	17.68 ± 1.84	0.48 ± 0.07	68.20 ± 7.27	66.00 ± 14.15	68.20 ± 7.27	3.62 ± 0.14	109.40 ± 13.34 ^a^	22.60 ± 1.30	88.25 ± 12.60	7.60 ± 0.62 ^a^	157.86 ± 1.88 ^ab^
2000AF	93.00 ± 9.14	18.12 ± 0.80	0.47 ± 0.05	58.20 ± 5.13	46.00 ± 5.18	58.20 ± 5.13	3.52 ± 0.08	135.20 ± 11.86	21.40 ± 1.30 ^a^	90.40 ± 3.09	7.63 ± 0.68 ^a^	159.52 ± 2.28 ^a^

Values expressed as mean ± S.E.M. (*p* < 0.05; one-way ANOVA). ^a^ Significantly different from control, ^b^ significantly different from 175AM. BUN: blood urea nitrogen, TC: total cholesterol, TG: triglycerides, AST: aspartate aminotransferase, ALT: alanine aminotransferase, ALP: alkaline phosphatase.

**Table 8:** Hematological parameters of male rats in acute toxicity study.

Group	Parameters													
	WBC	NEU	LYM	MON	EOS	BAS	RBC	HGB	HCT	MCV	MCH	MCHC	RDW	PLT
	(×10^3^/uL)	(%)	(%)	(%)	(%)	(%)	(×10^6^/uL)	(g/dL)	(%)	(fL)	(pg)	(g/dL)	(%)	(×10^3^/uL)
Control	4.94 ± 1.78	9.53 ± 0.93	82.26 ± 4.86	3.10 ± 0.46	1.00 ± 0.40	0.04 ± 0.04	7.51 ± 0.12	14.63 ± 0.16	43.88 ± 2.25	58.44 ± 1.66	19.70 ± 0.26	33.82 ± 1.07	15.52 ± 0.23	890.00 ± 84.23
175AM	6.59 ± 0.90	11.34 ± 1.27	81.64 ± 2.22	4.05 ± 0.57	1.43 ± 0.59	0.06 ± 0.04	7.58 ± 0.22	14.52 ± 0.28	44.28 ± 1.57	58.38 ± 0.96	19.16 ± 0.21	32.86 ± 0.55	17.14 ± 0.89	989.40 ± 92.31
550AM	5.78 ± 1.01	11.43 ± 1.20	80.82 ± 3.37	3.46 ± 0.34	0.78 ± 0.27	0.00 ± 0.00	7.31 ± 0.34	14.16 ± 0.69	42.14 ± 2.06	56.80 ± 0.93	19.32 ± 0.25	34.04 ± 0.59	17.15 ± 0.68	981.75 ± 67.71
2000AM	3.26 ± 0.87	10.75 ± 1.39	82.62 ± 2.31	2.60 ± 0.41	0.73 ± 0.06	0.00 ± 0.00	7.16 ± 0.37	14.74 ± 0.58	41.18 ± 2.45	57.44 ± 0.84	19.30 ± 0.17	33.68 ± 0.46	16.68 ± 0.84	1009.75 ± 81.54

Values expressed as mean ± S.E.M. (*p* < 0.05; one-way ANOVA). WBC: white blood cell (count), NEU: neutrophils, LYM: lymphocytes, MON: monocytes, EOS: eosinophils, BAS: basophils, RBC: red blood cell count (count), HGB: hemoglobin, HCT: hematocrit, MCV: mean corpuscular volume, MCH: mean corpuscular hemoglobin, MCHC: mean corpuscular hemoglobin concentration, RDW: red cell distribution width, PLT: platelet (count).

**Table 9:** Hematological parameters of female rats in acute toxicity study.

Group	Parameters													
	WBC	NEU	LYM	MON	EOS	BAS	RBC	HGB	HCT	MCV	MCH	MCHC	RDW	PLT
	(×10^3^/uL)	(%)	(%)	(%)	(%)	(%)	(×10^6^/uL)	(g/dL)	(%)	(fL)	(pg)	(g/dL)	(%)	(×10^3^/uL)
Control	4.86 ± 1.27	8.44 ± 1.89	85.92 ± 2.87	3.32 ± 0.84	2.30 ± 0.70	0.02 ± 0.02	7.30 ± 0.46	14.10 ± 0.86	43.75 ± 1.40	57.06 ± 1.16	19.32 ± 0.36	33.90 ± 1.11	15.56 ± 1.34	626.00 ± 120.40
175AF	5.84 ± 1.58	9.48 ± 1.74	85.88 ± 2.37	2.14 ± 0.40	1.98 ± 0.79	0.00 ± 0.00	7.73 ± 0.18	15.06 ± 0.33	44.74 ± 0.99	58.75 ± 1.52	20.02 ± 0.64	33.18 ± 0.41	14.42 ± 0.89	572.60 ± 155.47
550AF	3.34 ± 0.62	8.94 ± 1.12	86.22 ± 1.84	2.10 ± 0.64	0.88 ± 0.20	0.00 ± 0.00	7.90 ± 0.19	15.46 ± 0.40	45.14 ± 1.35	57.10 ± 0.85	19.56 ± 0.21	34.26 ± 0.18	16.40 ± 0.63	1109.20 ± 69.18 ^b^
2000AF	3.67 ± 0.99	9.22 ± 1.85	83.56 ± 3.79	2.60 ± 0.80	1.75 ± 0.57	0.00 ± 0.00	8.02 ± 0.28	15.42 ± 0.44	43.78 ± 1.03	56.44 ± 0.81	19.24 ± 0.12	34.12 ± 0.63	15.20 ± 0.34	862.60 ± 172.06

Values expressed as mean ± S.E.M. (*p* < 0.05; one-way ANOVA). ^b^ Significantly different from control. WBC: white blood cell (count), NEU: neutrophils, LYM: lymphocytes, MON: monocytes, EOS: eosinophils, BAS: basophils, RBC: red blood cell count (count), HGB: hemoglobin, HCT: hematocrit, MCV: mean corpuscular volume, MCH: mean corpuscular hemoglobin, MCHC: mean corpuscular hemoglobin concentration, RDW: red cell distribution width, PLT: platelet (count).

**Table 10:** Signs of toxicity and mortality of male rats in acute toxicity study.

Group	Effects		
	D/T	Mortality Latency (h)	Signs of Toxicity
Control	0/5	-	None
175AM	0/5	-	None
550AM	0/5	-	None
2000AM	0/5	-	None

D/T: number of deaths/total number treated. Mortality latency: time to death (in hours) following the oral administration. None: non-toxic signs during the observation period.

**Table 11:** Signs of toxicity and mortality of female rats in acute toxicity study.

Group	Effects		
	D/T	Mortality Latency (h)	Signs of Toxicity
Control	0/5	-	None
175AF	0/5	-	None
550AF	0/5	-	None
2000AF	0/5	-	None

D/T: number of deaths/total number treated. Mortality latency: time to death (in hours) following the oral administration. None: non-toxic signs during the observation period.

**Table 12:** Histopathological alterations of male rats in acute toxicity study.

Group	Liver	Kidney	Heart	Spleen	Lung	
	Mild HV/HD	N	N	N	N AVT	IP
Control	2/5	5/5	5/5	5/5	1/5	3/5
175AM	1/5	5/5	5/5	5/5	2/5	2/5
550AM	2/5	5/5	5/5	5/5	0/5	4/5
2000AM	0/5	5/5	5/5	5/5	0/5	4/5

The results are expressed as the number of rats with pathological findings/total number of rats. Liver: HV = hepatic vacuolation/HD = hepatic degeneration/N = normal (very mild lesion). Kidney: N = normal (some specimens show renal tubular autolysis in scattering areas, which could be caused by poor formalin fixation or delayed specimen collection). Spleen: N = normal (splenic congestion is demonstrated in some specimens). Heart: N = normal (some specimens show myocardial autolysis in scattering areas, which could be caused by poor formalin fixation or delayed specimen collection). Lung: N = normal/N AVT = normal with alveolar septal thickness /IP = interstitial pneumonitis (the histopathologic feature of the lungs shows varying severity degrees of alveolar edema, alveolar septal engorgement, alveolar hemorrhage, and inflammatory cell infiltration to pulmonary parenchyma).

**Table 13:** Histopathological alterations of female rats in acute toxicity study.

Group	Liver	Kidney	Heart	Spleen	Lung
	Mild HV/HD	N	N	N	IP
Control	0/5	5/5	5/5	5/5	5/5
175AF	0/5	5/5	5/5	5/5	5/5
550AF	1/5	5/5	5/5	5/5	5/5
2000AF	0/5	5/5	5/5	5/5	4/5

The results are expressed as the number of rats with pathological findings/total number of rats. Liver: HV = hepatic vacuolation/HD = hepatic degeneration/N = normal (very mild lesion). Kidney: N = normal (some specimens show renal tubular autolysis in scattering areas, which could be caused by poor formalin fixation or delayed specimen collection). Spleen: N = normal (splenic congestion is demonstrated in some specimens). Heart: N = normal (some specimens show myocardial autolysis in scattering areas, which could be caused by poor formalin fixation or delayed specimen collection). Lung: N = normal/IP = interstitial pneumonitis (the histopathologic features of the lungs show varying severity degrees of alveolar edema, alveolar septal engorgement, alveolar hemorrhage, and inflammatory cell infiltration to pulmonary parenchyma).

**Table 14:** Body weight gain of male rats in 28-day repeated dose sub-acute toxicity study.

Group	Weight Gain (g) from Week 0					
	Week 1	Week 2	Week 3	Week 4	Week 5	Week 6
Control	206 ± 8.55	236 ± 6.22	254 ± 5.12 ^mn^	273 ± 8.66 ^mno^	-	-
250RM	208 ± 6.75	230 ± 9.52 ^m^	256 ± 8.18 ^mn^	288 ± 12.80 ^mno^	-	-
500RM	221 ± 13.39	259 ± 9.82 ^m^	278 ± 10.67 ^mn^	308 ± 11.90 ^mno^	-	-
1000RM	219 ± 7.58	236 ± 7.22 ^m^	265 ± 11.79 ^mn^	298 ± 12.80 ^mno^	-	-
Satellite						
S-Control	228 ± 9.45	262 ± 11.94 ^m^	277 ± 15.37 ^m^	306 ± 16.05 ^mno^	338 ± 19.73 ^mnop^	378 ± 21.84 ^mnopq^
S-1000RM	225 ± 6.85	247 ± 4.18	263 ± 8.40 ^m^	295 ± 6.61 ^mn^	315 ± 5.59 ^mno^	366 ± 12.42 ^mnopq^

Values expressed as mean ± S.E.M. (*p* < 0.05; two-way ANOVA; Tukey Method). ^m^ Significantly different from week 0, ^n^ significantly different from week 1, ^o^ significantly different from week 2, ^p^ significantly different from week 3, ^q^ significantly different from week 4.

**Table 15:** Body weight gain of female rats in 28-day repeated dose sub-acute toxicity study.

Group	Weight Gain (g) from Week 0					
	Week 1	Week 2	Week 3	Week 4	Week 5	Week 6
Control	163 ± 2.85	169 ± 4.47	187 ± 4.18 ^mn^	209 ± 4.47 ^mno^	-	-
250RF	166 ± 3.26	174 ± 2.74	188 ± 7.20 ^mn^	203 ± 4.87 ^mno^	-	-
500RF	166 ± 5.42	172 ± 4.87	182 ± 3.79 ^m^	200 ± 4.68 ^mno^	-	-
1000RF	168 ± 2.85	171 ± 1.12	187 ± 2.24 ^mn^	203 ± 4.18 ^mno^	-	-
Satellite						
S-Control	165 ± 5.86	171 ± 5.70	189 ± 6.94 ^mn^	201 ± 8.73 ^mno^	210 ± 5.27 ^mnop^	228 ± 5.00 ^mnopq^
S-1000RF	166 ± 2.09	169 ± 3.26	187 ± 4.54 ^mn^	201 ± 5.70 ^mno^	212 ± 5.76 ^mno^	226 ± 6.47 ^mnopq^

Values expressed as mean ± S.E.M. (*p* < 0.05; two-way ANOVA; Tukey Method). ^m^ Significantly different from week 0, ^n^ significantly different from week 1, ^o^ significantly different from week 2, ^p^ significantly different from week 3, ^q^ significantly different from week 4.

**Table 16:** Weight gain of male rats in 28-day repeated dose sub-acute toxicity study (satellite).

Group	Weight Gain (g) from Week 0					
	Week 1	Week 2	Week 3	Week 4	Week 5	Week 6
Control	30 ± 5.86	48 ± 4.87 ^n^	70 ± 4.08 ^no^	99 ± 7.59 ^nop^	-	-
250RM	22 ± 3.35	48 ± 4.18 ^n^	76 ± 6.40 ^no^	111 ± 8.29 ^nop^	-	-
500RM	29 ± 2.76	48 ± 5.53 ^n^	78 ± 3.73 ^no^	116 ± 6.40 ^nop^	-	-
1000RM	14 ± 4.33	43 ± 6.87 ^n^	75 ± 11.06 ^no^	109 ± 13.82 ^nop^	-	-
Satellite						
S-Control	34 ± 4.81	49 ± 6.22	78 ± 7.62 ^no^	110 ± 10.75 ^nop^	150 ± 12.87 ^nopq^	160 ± 17.41 ^nopq^
S-1000RM	22 ± 5.18	38 ± 6.75	70 ± 5.00 ^no^	90 ± 8.48 ^no^	141 ± 9.91 ^nopq^	155 ± 9.19 ^nopq^

Values expressed as mean ± S.E.M. (*p* < 0.05; two-way ANOVA; Tukey Method). ^n^ Significantly different from week 1, ^o^ significantly different from week 2, ^p^ significantly different from week 3, ^q^ significantly different from week 4.

**Table 17:** Weight gain of female rats in 28-day repeated dose sub-acute toxicity study (satellite).

Group	Weight Gain (g) from Week 0					
	Week 1	Week 2	Week 3	Week 4	Week 5	Week 6
Control	6 ± 3.26	24 ± 3.26 ^n^	46 ± 2.74 ^no^	51 ± 3.26 ^no^	-	-
250RF	8 ± 2.85	22 ± 4.54 ^n^	37 ± 2.85 ^no^	45 ± 5.59 ^no^	-	-
500RF	6 ± 3.71	16 ± 2.74	34 ± 4.81 ^no^	49 ± 4.93 ^nop^	-	-
1000RF	3 ± 3.35	19 ± 4.11 ^n^	35 ± 3.54 ^no^	43 ± 3.79 ^no^	-	-
Satellite						
S-Control	6 ± 2.09	24 ± 2.74 ^n^	36 ± 4.81 ^no^	50 ± 4.08 ^nop^	68 ± 3.73 ^nopq^	66 ± 1.44 ^nopq^
S-1000RF	3 ± 2.24	21 ± 4.47 ^n^	35 ± 3.95 ^no^	46 ± 3.71 ^no^	60 ± 5.00 ^nopq^	67 ± 5.76 ^nopq^

Values expressed as mean ± S.E.M. (*p* < 0.05; two-way ANOVA; Tukey Method). ^n^ Significantly different from week 1, ^o^ significantly different from week 2, ^p^ significantly different from week 3, ^q^ significantly different from week 4.

**Table 18:** Food consumption of male rats in 28-day repeated dose toxicity study.

Group	Average Food Consumption (g/rat/day)					
	Week 1	Week 2	Week 3	Week 4	Week 5	Week 6
Control	16.72 ± 0.49	14.48 ± 0.74	16.91 ± 1.31 ^o^	17.76 ± 0.45 ^o^	-	-
250RM	14.85 ± 0.20 ^a^	16.66 ± 0.25 ^an^	17.27 ± 0.10 ^n^	18.86 ± 0.26 ^nop^	-	-
500RM	15.07 ± 1.49	17.33 ± 0.17 ^an^	17.91 ± 0.46 ^n^	17.88 ± 0.32 ^n^	-	-
1000RM	11.36 ± 0.26 ^abc^	16.61 ± 0.61 ^an^	17.52 ± 0.15 ^n^	18.32 ± 0.63 ^no^	-	-
Satellite						
S-Control	18.03 ± 0.42	17.33 ± 1.05	17.75 ± 0.91	18.26 ± 1.06	23.37 ± 1.70 ^nopq^	24.90 ± 1.77 ^nopqr^
S-1000RM	14.29 ± 0.10 ^a^	14.91 ± 0.42	16.39 ± 0.29 ^no^	16.86 ± 0.08 ^no^	22.32 ± 0.05 ^nopq^	22.76 ± 0.16 ^nopq^

Values expressed as mean ± S.E.M. (*p* < 0.05; two-way ANOVA; Tukey Method). ^a^ Significantly different from the control group in that week. ^b^ Significantly different from the 250RM group in that week. ^c^ Significantly different from the 500RM in that week. ^n^ Significantly different from week 1, ^o^ significantly different from week 2, ^p^ significantly different from week 3, ^q^ significantly different from week 4, ^r^ significantly different from week 5.

**Table 19:** Food consumption of female rats in 28-day repeated dose toxicity study.

Group	Average Food Consumption (g/rat/day)					
	Week 1	Week 2	Week 3	Week 4	Week 5	Week 6
Control	12.15 ± 0.05	13.02 ± 0.33 ^n^	13.69 ± 0.09 ^n^	13.32 ± 0.31 ^n^	-	-
250RF	11.48 ± 0.13	12.77 ± 0.43 ^n^	13.28 ± 0.12 ^n^	13.80 ± 0.27 ^no^	-	-
500RF	11.25 ± 0.21 ^a^	11.59 ± 0.07 ^ab^	12.83 ± 0.13 ^ano^	11.52 ± 0.15 ^abp^	-	-
1000RF	10.92 ± 0.22 ^a^	11.73 ± 0.01 ^abn^	12.92 ± 0.29 ^ano^	12.12 ± 0.01 ^abnp^	-	-
Satellite						
S-Control	11.76 ± 0.64	12.15 ± 1.11	12.82 ± 1.21 ^n^	14.02 ± 0.40 ^nop^	15.82 ± 0.62 ^nopq^	16.43 ± 0.83 ^nopq^
S-1000RF	11.55 ± 0.11	11.98 ± 0.53	12.18 ± 0.11	12.67 ± 0.06 ^n^	15.91 ± 0.17 ^nopq^	16.52 ± 0.38 ^nopq^

Values expressed as mean ± S.E.M. (*p* < 0.05; two-way ANOVA; Tukey Method). ^a^ Significantly different from the control group in that week. ^b^ Significantly different from the 250RF group in that week. ^n^ Significantly different from week 1, ^o^ significantly different from week 2, ^p^ significantly different from week 3, ^q^ significantly different from week 4.

**Table 20:** Relative organ weight of male rats in 28-day repeated dose toxicity study.

Group	ROW (g per 100 g Body Weight)										
	Liver	Kidneys	Heart	Spleen	Lung	Adrenal	Thymus	Brain	Testes	Epididymis	Seminal Vesicles
						Gland	Gland				Coagulating Glands
Control	3.57 ± 0.18	0.80 ± 0.03	0.28 ± 0.01	0.20 ± 0.01	0.41 ± 0.01	0.03 ± 0.00	0.02 ± 0.00	0.63 ± 0.02	1.02 ± 0.03	0.34 ± 0.01	0.32 ± 0.03
250RM	3.73 ± 0.22	0.80 ± 0.05	0.27 ± 0.01	0.20 ± 0.01	0.40 ± 0.02	0.02 ± 0.00	0.01 ± 0.00	0.63 ± 0.03	0.95 ± 0.03	0.30 ± 0.01	0.29 ± 0.04
500RM	3.66 ± 0.20	0.79 ± 0.03	0.27 ± 0.01	0.21 ± 0.02	0.42 ± 0.03	0.02 ± 0.00	0.01 ± 0.00	0.58 ± 0.02	0.94 ± 0.03	0.32 ± 0.03	0.30 ± 0.03
1000RM	4.40 ± 0.14 ^ac^	0.86 ± 0.02	0.27 ± 0.01	0.24 ± 0.01	0.43 ± 0.03	0.03 ± 0.00	0.01 ± 0.00	0.53 ± 0.07	0.99 ± 0.05	0.34 ± 0.02	0.29 ± 0.01
Satellite											
S-Control	3.44 ± 0.07	0.77 ± 0.04	0.28 ± 0.01	0.21 ± 0.02	0.41 ± 0.04	0.02 ± 0.00	0.01 ± 0.00	0.54 ± 0.03	0.88 ± 0.07	0.37 ± 0.02	0.23 ± 0.02
S-1000RM	3.88 ± 0.14 ^A^	0.85 ± 0.01	0.29 ± 0.01	0.23 ± 0.01	0.46 ± 0.04	0.03 ± 0.00	0.02 ± 0.00	0.53 ± 0.02	0.97 ± 0.03	0.44 ± 0.05	0.30 ± 0.02 ^A^

Values expressed as mean ± S.E.M. (*p* < 0.05; one-way ANOVA). ^a^ Significantly different from control, ^c^ significantly different from 500RM, ^A^ significantly different from S-Control.

**Table 21:** Relative organ weight of female rats in 28-day repeated dose toxicity study.

Group	ROW (g per 100 g Body Weight)									
	Liver	Kidneys	Heart	Spleen	Lung	Adrenal Gland	Thymus Gland	Brain	Ovary	Uterus
Control	4.35 ± 0.16	0.93 ± 0.03	0.32 ± 0.01	0.27 ± 0.01	0.51 ± 0.02	0.04 ± 0.00	0.01 ± 0.00	0.87 ± 0.02	0.07 ± 0.01	0.21 ± 0.03
250RF	3.88 ± 0.22	0.96 ± 0.03	0.33 ± 0.01	0.27 ± 0.02	0.49 ± 0.01	0.05 ± 0.00	0.02 ± 0.00	0.85 ± 0.03	0.10 ± 0.02	0.20 ± 0.02
500RF	3.37 ± 0.12 ^a^	0.81 ± 0.04 ^ab^	0.32 ± 0.01	0.26 ± 0.01	0.59 ± 0.06	0.04 ± 0.00	0.02 ± 0.00	0.82 ± 0.02	0.08 ± 0.01	0.29 ± 0.05
1000RF	3.46 ± 0.13 ^a^	0.82 ± 0.01 ^ab^	0.29 ± 0.01 ^b^	0.26 ± 0.00	0.50 ± 0.03	0.04 ± 0.01	0.02 ± 0.00	0.82 ± 0.01	0.08 ± 0.01	0.21 ± 0.02
Satellite										
S-Control	3.54 ± 0.11	0.87 ± 0.02	0.32 ± 0.00	0.27 ± 0.01	0.52 ± 0.07	0.05 ± 0.00	0.02 ± 0.00	0.83 ± 0.01	0.10 ± 0.01	0.27 ± 0.03
S-1000RF	3.77 ± 0.10	0.86 ± 0.03	0.32 ± 0.00	0.27 ± 0.01	0.53 ± 0.03	0.04 ± 0.00	0.02 ± 0.00	0.85 ± 0.03	0.11 ± 0.01	0.27 ± 0.03

Values expressed as mean ± S.E.M. (*p* < 0.05; one-way ANOVA). ^a^ Significantly different from control, ^b^ significantly different from 250RM.

**Table 22:** Biochemical parameters of male rats in 28-day repeated dose toxicity study.

Group	Parameters											
	Blood Sugar	BUN	Creatinine	TC	TG	Total Protein	Albumin	AST	ALT	ALP	Potassium	Sodium
	(mg/dL)	(mg/dL)	(mg/dL)	(mg/dL)	(mg/dL)	(g/dL)	(g/dL)	(U/L)	(U/L)	(U/L)	(mmol/L)	(mEq/L)
Control	186.20 ± 26.55	26.10 ± 1.93	0.47 ± 0.03	71.60 ± 2.02	117.00 ± 12.85	6.12 ± 0.21	3.48 ± 0.14	104.67 ± 25.10	29.60 ± 3.56	121.80 ± 13.34	6.68 ± 0.36	137.90 ± 1.36
250RM	222.75 ± 57.16	25.73 ± 2.29	0.50 ± 0.10	66.33 ± 7.88	157.25 ± 10.25	6.38 ± 0.29	3.73 ± 0.33	74.33 ± 1.63	34.25 ± 4.20	117.50 ± 19.81	7.11 ± 0.58	139.78 ± 0.64
500RM	239.80 ± 36.12	23.74 ± 1.28	0.49 ± 0.05	64.20 ± 5.80	120.40 ± 26.54	6.08 ± 0.09	3.48 ± 0.05	90.20 ± 8.90	34.80 ± 3.93	122.00 ± 10.41	7.19 ± 0.40	140.18 ± 2.15
1000RM	211.00 ± 42.37	25.48 ± 1.54	0.44 ± 0.04	70.20 ± 6.20	145.60 ± 9.75	6.52 ± 0.07	3.76 ± 0.10	86.67 ± 17.33	37.20 ± 5.21	132.80 ± 9.30	6.61 ± 0.26	140.10 ± 1.40
Satellite												
S-Control	172.60 ± 16.78	18.46 ± 0.61	0.52 ± 0.06	67.60 ± 4.13	102.20 ± 15.38	6.14 ± 0.10	3.42 ± 0.09	71.60 ± 9.05	27.80 ± 2.90	106.40 ± 9.10	6.30 ± 0.24	136.26 ± 0.13
S-1000RM	230.20 ± 14.73	17.46 ± 0.93	0.42 ± 0.03	73.00 ± 2.74	89.80 ± 6.81	6.26 ± 0.06	3.42 ± 0.07	71.80 ± 4.28	29.60 ± 2.20	104.80 ± 9.74	6.11 ± 0.27	136.50 ± 0.20

Values expressed as mean ± S.E.M. (*p* < 0.05; one-way ANOVA). BUN: blood urea nitrogen, TC: total cholesterol, TG: triglycerides, AST: aspartate aminotransferase, ALT: alanine aminotransferase, ALP: alkaline phosphatase.

**Table 23:** Biochemical parameters of female rats in 28-day repeated dose toxicity study.

Group	Parameters											
	Blood Sugar	BUN	Creatinine	TC	TG	Total Protein	Albumin	AST	ALT	ALP	Potassium	Sodium
	(mg/dL)	(mg/dL)	(mg/dL)	(mg/dL)	(mg/dL)	(g/dL)	(g/dL)	(U/L)	(U/L)	(U/L)	(mmol/L)	(mEq/L)
Control	134.25 ± 20.34	24.53 ± 2.32	0.35 ± 0.03	75.75 ± 11.50	105.00 ± 18.12	6.88 ± 0.25	4.08 ± 0.28	121.75 ± 20.83	35.25 ± 7.55	82.75 ± 13.82	6.41 ± 0.24	140.90 ± 1.01
250RF	133.50 ± 27.47	21.08 ± 1.44	0.43 ± 0.12	57.67 ± 8.26	76.75 ± 19.76	7.25 ± 0.70	4.20 ± 0.38	121.00 ± 17.37	37.00 ± 3.74	63.00 ± 6.41	7.02 ± 0.82	140.63 ± 0.91
500RF	90.00 ± 16.57	19.30 ± 0.46	0.52 ± 0.06	61.67 ± 3.19	50.67 ± 6.79	6.90 ± 0.19	4.27 ± 0.15	150.00 ± 27.28	37.00 ± 5.52	66.33 ± 5.76	6.26 ± 0.59	142.50 ± 1.06
1000RF	118.50 ± 14.72	20.73 ± 1.33	0.45 ± 0.04	62.25 ± 8.02	66.00 ± 10.04	6.68 ± 0.12	3.83 ± 0.09	100.25 ± 7.14	25.75 ± 1.66	71.50 ± 5.69	6.42 ± 0.36	141.13 ± 0.16
Satellite												
S-Control	107.75 ± 7.68	16.93 ± 0.88	0.46 ± 0.03	53.50 ± 4.18	48.50 ± 8.86	6.60 ± 0.16	3.65 ± 0.15	100.50 ± 8.14	24.75 ± 1.52	56.75 ± 1.72	7.12 ± 0.34	136.25 ± 0.56
S-1000RF	104.33 ± 11.28	17.40 ± 0.53	0.49 ± 0.01	62.75 ± 7.20	57.00 ± 9.58	6.75 ± 0.12	3.93 ± 0.11	88.25 ± 8.49	24.00 ± 1.25	57.00 ± 4.83	6.45 ± 0.48	136.75 ± 0.54

Values expressed as mean ± S.E.M. (*p* < 0.05; one-way ANOVA). BUN: blood urea nitrogen, TC: total cholesterol, TG: triglycerides, AST: aspartate aminotransferase, ALT: alanine aminotransferase, ALP: alkaline phosphatase.

**Table 24:** Hematological parameters of male rats in 28-day repeated dose toxicity study.

Group	Parameters													
	WBC	NEU	LYM	MON	EOS	BAS	RBC	HGB	HCT	MCV	MCH	MCHC	RDW	PLT
	(×10^3^/uL)	(%)	(%)	(%)	(%)	(%)	(×10^6^/uL)	(g/dL)	(%)	(fL)	(pg)	(g/dL)	(%)	(×10^3^/uL)
Control	6.24 ± 1.41	10.32 ± 0.41	86.18 ± 1.09	1.94 ± 0.36	1.32 ± 0.42	0.24 ± 0.13	8.22 ± 0.27	15.42 ± 0.43	46.58 ± 1.65	56.66 ± 1.20	18.78 ± 0.32	33.16 ± 0.46	19.14 ± 0.64	808.33 ± 110.09
250RM	8.60 ± 2.31	9.70 ± 0.64	85.28 ± 1.43	2.15 ± 0.22	1.07 ± 0.36	0.27 ± 0.15	8.30 ± 0.29	15.40 ± 0.65	48.10 ± 2.04	57.93 ± 0.54	18.55 ± 0.17	32.03 ± 0.31	19.73 ± 0.10	899.25 ± 59.99
500RM	7.90 ± 2.01	12.46 ± 2.37	84.64 ± 2.00	1.58 ± 0.48	0.86 ± 0.34	0.13 ± 0.11	8.28 ± 0.19	15.74 ± 0.33	50.22 ± 1.29	60.64 ± 1.12	19.00 ± 0.19	31.36 ± 0.34	19.44 ± 0.29	847.00 ± 52.07
1000RM	6.01 ± 1.07	9.32 ± 0.47	86.64 ± 0.69	2.00 ± 0.50	0.75 ± 0.21	0.50 ± 0.22	8.49 ± 0.15	15.80 ± 0.33	50.76 ± 1.15	59.82 ± 1.11	18.62 ± 0.24	31.14 ± 0.26	19.62 ± 0.39	801.40 ± 59.68
Satellite														
S-Control	7.23 ± 1.61	8.48 ± 0.54	88.80 ± 0.27	1.38 ± 0.60	0.86 ± 0.12	0.30 ± 0.22	9.17 ± 0.17	16.40 ± 0.11	52.84 ± 0.39	57.68 ± 1.05	17.90 ± 0.35	31.02 ± 0.10	21.12 ± 0.22	1003.60 ± 96.05
S-1000RM	9.57 ± 1.09	7.80 ± 0.52	89.36 ± 0.31	1.64 ± 0.57	0.98 ± 0.11	0.22 ± 0.22	8.75 ± 0.23	16.08 ± 0.20	52.42 ± 0.52	60.00 ± 1.36	18.40 ± 0.36	30.68 ± 0.17	20.74 ± 0.25	1002.00 ± 32.33

Values expressed as mean ± S.E.M. (*p* < 0.05; one-way ANOVA). WBC: white blood cell (count), NEU: neutrophils, LYM: lymphocytes, MON: monocytes, EOS: eosinophils, BAS: basophils, RBC: red blood cell count (count), HGB: hemoglobin, HCT: hematocrit, MCV: mean corpuscular volume, MCH: mean corpuscular hemoglobin, MCHC: mean corpuscular hemoglobin concentration, RDW: red cell distribution width, PLT: platelet (count).

**Table 25:** Hematological parameters of female rats in 28-day repeated dose toxicity study.

Group	Parameters													
	WBC	NEU	LYM	MON	EOS	BAS	RBC	HGB	HCT	MCV	MCH	MCHC	RDW	PLT
	(×10^3^/uL)	(%)	(%)	(%)	(%)	(%)	(×10^6^/uL)	(g/dL)	(%)	(fL)	(pg)	(g/dL)	(%)	(×10^3^/uL)
Control	5.03 ± 1.23	11.53 ± 1.02	84.40 ± 1.07	2.68 ± 0.62	0.55 ± 0.18	0.40 ± 0.19	7.90 ± 0.25	15.18 ± 0.46	49.05 ± 2.05	62.08 ± 0.87	19.23 ± 0.13	30.98 ± 0.46	18.70 ± 0.77	744.67 ± 81.92
250RF	5.49 ± 1.48	9.95 ± 1.95	84.33 ± 3.27	1.65 ± 0.70	1.80 ± 0.82	1.05 ± 0.49	7.98 ± 0.39	15.03 ± 0.63	47.68 ± 1.98	59.85 ± 1.36	18.88 ± 0.52	31.53 ± 0.17	19.38 ± 0.57	694.00 ± 43.62
500RF	6.11 ± 0.97	9.43 ± 1.21	86.00 ± 1.85	1.98 ± 0.36	0.93 ± 0.18	0.95 ± 0.35	8.12 ± 0.05	16.05 ± 0.25	50.03 ± 0.63	61.63 ± 0.85	19.75 ± 0.35	32.10 ± 0.57	19.53 ± 0.62	820.50 ± 99.40
1000RF	6.66 ± 1.13	9.96 ± 0.71	86.28 ± 1.37	1.82 ± 0.40	1.52 ± 0.56	0.42 ± 0.20	8.16 ± 0.06	15.78 ± 0.21	50.80 ± 0.95	62.24 ± 1.03	19.32 ± 0.25	31.06 ± 0.19	19.64 ± 0.33	822.80 ± 71.20
Satellite														
S-Control	7.45 ± 0.84	7.92 ± 0.40	88.04 ± 0.95	2.54 ± 0.45	1.44 ± 0.54	0.06 ± 0.07	8.76 ± 0.20	16.68 ± 0.29	53.86 ± 1.39	61.48 ± 0.67	19.04 ± 0.26	30.98 ± 0.30	21.88 ± 0.43	844.20 ± 72.02
S-1000RF	5.91 ± 0.78	8.04 ± 0.63	87.42 ± 0.57	2.40 ± 0.36	1.44 ± 0.20	0.35 ± 0.21	8.38 ± 0.20	15.88 ± 0.37	53.08 ± 1.75	63.30 ± 1.39	18.94 ± 0.19	29.96 ± 0.40	20.86 ± 0.28	912.40 ± 50.99

Values expressed as mean ± S.E.M. (*p* < 0.05; one-way ANOVA). WBC: white blood cell (count), NEU: neutrophils, LYM: lymphocytes, MON: monocytes, EOS: eosinophils, BAS: basophils, RBC: red blood cell count (count), HGB: hemoglobin, HCT: hematocrit, MCV: mean corpuscular volume, MCH: mean corpuscular hemoglobin, MCHC: mean corpuscular hemoglobin concentration, RDW: red cell distribution width, PLT: platelet (count).

**Table 26:** Signs of toxicity and mortality of male rats in 28-day repeated dose toxicity study.

Group	Effects		
	D/T	Mortality Latency (h)	Signs of Toxicity
Control	0/5	-	None
250RM	0/5	-	None
500RM	0/5	-	None
1000RM	0/5	-	None
Satellite			
S-Control	0/5	-	None
S-1000RM	0/5	-	None

D/T: number of deaths/total number treated. Mortality latency: time to death (in hours) following the oral administration. None: non-toxic signs during the observation period.

**Table 27:** Signs of toxicity and mortality of female rats in 28-day repeated dose toxicity study.

Group	Effects		
	D/T	Mortality Latency (h)	Signs of Toxicity
Control	0/5	-	None
250RF	0/5	-	None
500RF	0/5	-	None
1000RF	0/5	-	None
Satellite			
S-Control	0/5	-	None
S-1000RF	0/5	-	None

D/T: number of deaths/total number treated. Mortality latency: time to death (in hours) following the oral administration. None: non-toxic signs during the observation period.

**Table 28:** Histopathological of male rats in 28-day repeated dose toxicity study.

Group	Liver	Kidney	Heart	Spleen	Lung		
	Mild HV/HD	N	N	N	IP	N AVT	N AVHy
Control	3/5	5/5	5/5	5/5	5/5	-	-
250RM	5/5	5/5	5/5	5/5	4/5	-	-
500RM	4/5	5/5	5/5	5/5	3/5	1/5	-
1000RM	4/5	5/5	5/5	5/5	5/5	-	-
Satellite							
S-Control	1/5	5/5	5/5	5/5	-	5/5	-
S-1000RM	1/5	5/5	5/5	5/5	1/5	3/5	1/5

The results are expressed as the number of rats with pathological findings/total number of rats. Liver: HV = hepatic vacuolation/HD = hepatic degeneration/N = normal (very mild lesion). Kidney: N = normal (some specimens show renal tubular autolysis in scattering areas, which could be caused by poor formalin fixation or delayed specimen collection). Spleen: N = normal (splenic congestion is demonstrated in some specimens). Heart: N = normal (some specimens show myocardial autolysis in scattering areas, which could be caused by poor formalin fixation or delayed specimen collection). Lung: N = normal/N AVT = normal with alveolar septal thickness/N AVHy = normal with alveolar hyperemia/IP = interstitial pneumonitis (the histopathologic feature of the lungs shows varying severity degrees of alveolar edema, alveolar septal engorgement, alveolar hemorrhage, and inflammatory cell infiltration to pulmonary parenchyma).

**Table 29:** Histopathological of female rats in 28-day repeated dose toxicity study.

Group	Liver		Kidney	Heart	Spleen	Lung	
	Mild HV/HD	Diffuse HV	N	N	N	IP	N AVT
Control	4/5	-	5/5	5/5	5/5	4/5	1/5
250RF	4/5	1/5	5/5	5/5	5/5	4/5	-
500RF	5/5	-	5/5	5/5	5/5	1/5	1/5
1000RF	5/5	-	5/5	5/5	5/5	4/5	1/5
Satellite							
S-Control	1/5	-	5/5	5/5	5/5	3/5	5/5
S-1000RF	1/5	-	5/5	5/5	5/5	4/5	-

The results are expressed as the number of rats with pathological findings/total number of rats. Liver: HV = hepatic vacuolation/HD = hepatic degeneration/N = normal (very mild lesion). Kidney: N = normal (some specimens show renal tubular autolysis in scattering areas, which could be caused by poor formalin fixation or delayed specimen collection). Spleen: N = normal (splenic congestion is demonstrated in some specimens). Heart: N = normal (some specimens show myocardial autolysis in scattering areas, which could be caused by poor formalin fixation or delayed specimen collection). Lung: N = normal/N AVT = normal with alveolar septal thickness/IP = interstitial pneumonitis (the histopathologic features of the lungs show varying severity degrees of alveolar edema, alveolar septal engorgement, alveolar hemorrhage, and inflammatory cell infiltration to pulmonary parenchyma).

## Data Availability

The original contributions presented in this study are included in the article, and further inquiries can be directed to the corresponding authors.
